# Twist1-induced miR-199a-3p promotes liver fibrosis by suppressing caveolin-2 and activating TGF-β pathway

**DOI:** 10.1038/s41392-020-0169-z

**Published:** 2020-06-05

**Authors:** Xiaoxue Yang, Liping Ma, Rong Wei, Tinghong Ye, JianKang Zhou, Maoyao Wen, Ruoting Men, Rami I. Aqeilan, Yong Peng, Li Yang

**Affiliations:** 1 Department of Gastroenterology & Hepatology, West China Hospital, Sichuan University, Chengdu, 610041 China; 2 State Key Laboratory of Biotherapy and Cancer Centre, West China Hospital, Sichuan University, and Collaborative Innovation Centre for Biotherapy, Chengdu, 610041 China; 3 School of Bioscience and Technology, Chengdu medical college, Chengdu, 610500 China; 4 Department of Immunology & Cancer Research, Hebrew University-Hadassah Medical School, Jerusalem, Israel

**Keywords:** Non-coding RNAs, Gastrointestinal diseases

## Abstract

The activation of hepatic stellate cells (HSCs) participates in liver fibrosis, and emerging evidences indicate that microRNAs (miRNAs) are abnormally expressed during HSC activation. However, the potential roles of miRNAs in liver fibrosis still remain elusive. Therefore, this study aimed to investigate the role of miR-199a-3p in liver fibrosis and its underlying mechanism. We found that miR-199a-3p expression was dramatically upregulated during HSC activation in vitro, and during liver fibrogenesis in CCl_4_-treated rats, and its liver expression was increased in the patients with cirrhosis. By the luciferase assay and RT-qPCR, we revealed that the expression of miR-199a-3p in HSCs was driven by the transcription factor Twist1 which could be further induced by TGF-β treatment. Functional studies showed that inhibition of miR-199a-3p in both human LX2 cells and rat HSCs significantly decreased the expression of fibrotic markers, such as fibronectin and connective tissue growth factor (CTGF), whereas the forced expression of miR-199a-3p exhibited opposite effects, demonstrating the role of miR-199a-3p in promoting HSC activation. Mechanistically, miR-199a-3p plays an important role in TGF-β signalling pathway activation through targeting CAV2 that negatively regulates the expression of transforming growth factor-beta receptor type I (TGFβRI). Importantly, administration of antagomiR-199a-3p in the CCl_4_-treated mice significantly ameliorated hepatic fibrosis. In conclusion, Twist1-induced miR-199a-3p mediates the activation of HSCs by suppressing CAV2 expression and subsequently increasing TGFβRI expression to promote TGF-β pathway. Our findings highlight the therapeutic potential of miR-199a-3p for hepatic fibrosis.

## Introduction

Liver fibrosis is defined as excess deposition of extracellular matrix (ECM) in response to various liver damages and ultimately progresses to decompensated cirrhosis or hepatocellular carcinoma (HCC) with limited therapeutic options.^1,2^ Activated hepatic stellate cells (HSCs) are considered as the key cell type driving liver fibrosis.^3^ Upon exposure to persistent liver injury, quiescent HSCs transdifferentiate into proliferative and contractile myofibroblast-like cells, with the upregulation of α-smooth muscle actin (α-SMA) and collagen type1-α1 (COL1α1).^4^ Activated HSCs release pro-fibrogenic factors, including transforming growth factor-β (TGF-β), connective tissue growth factor (CTGF), fibronectin (FN) and tissue inhibitor of metalloproteinases, which drive the deposition of ECM.^4^ Given the lack of curing or reversing treatment for liver fibrosis currently, new insights into the molecular mechanisms controlling HSC activation are essential to discover new effective therapeutic strategies for liver fibrosis.

Three caveolins, caveolin-1 (CAV1), caveolin-2 (CAV2) and caveolin-3 (CAV3), have been identified in mammalian cells. CAV1 and CAV2 are usually co-expressed in most tissues and form homo-oligomers or hetero-oligomers complex in many cell types, including adipocytes, endothelial cells and fibroblasts, while CAV3 is exclusively expressed in muscle cells.^5^ Over the past decades, CAV1 has emerged as an important regulator of various liver diseases such as liver steatosis, fibrosis, and HCC.^6–10^ For example, CAV1-related selective autophagy is reported to promote liver sinusoidal endothelial cell defenestration, thus initiating liver fibrosis.^11^ CAV1 was also demonstrated to enhance HCC tumourigenesis and metastasis through activating the NF-κB pathway.^12^ Although CAV2 has a similar expression pattern to CAV1, its role in liver diseases remains elusive.

MicroRNAs (miRNAs) represent a subclass of small non-coding RNAs with about 22 nucleotides in length, regulating gene expression via inhibition of translation or stability of the target sequences.^13^ Accumulating evidences have established the involvement of certain miRNAs in the initiation and progression of liver fibrosis. For instance, miR-378a-3p was demonstrated to limit activation of HSCs and liver fibrosis by targeting Gli3 expression.^14^ Conversely, miR-214 promotes HSC activation and liver fibrosis by suppressing Sufu expression.^15^ In addition, multiple miRNAs (i.e. miR-122, miR-101, miR-133a, miR-221/222, miR-181b and miR-19b) participate in controlling HSC activation and liver fibrosis.^16–21^

In this study, we identified the fine regulation of miR-199a-3p in HSC activation and liver fibrosis. MiR-199a-3p, induced by Twist1 and TGF-β, positively regulates TGF-β signalling pathway by inhibiting CAV2-mediated TGF-β type I (TGFβRI) degradation. More importantly, silencing miR-199a-3p by antagomiR in vivo mitigated murine hepatic fibrosis induced by CCl_4_, highlighting its role as a promising target to slow and even reverse liver fibrosis.

## Results

### miR-199a-3p expression is increased during HSC activation

**Fig. 1:**
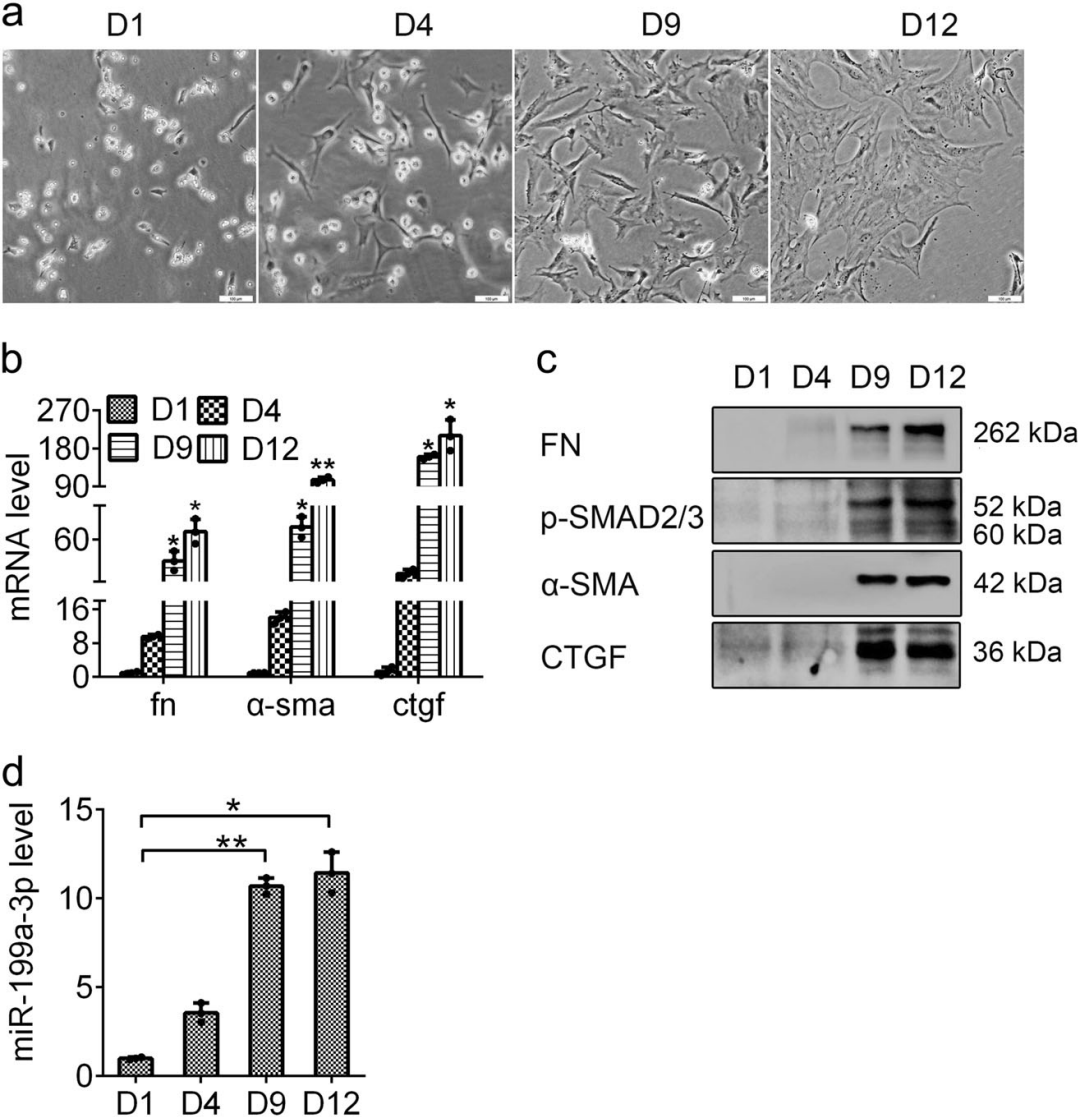
miR-199a-3p is upregulated in activated HSCs. **a** Morphological images of rat primary HSCs cultured for certain time in vitro at ×100 magnification. **b** Upregulated mRNA and **c** protein expression of fibrotic markers including FN, α-SMA, CTGF and p-SMAD2/3 during HSC activation. **d** Measurement of miR-199a-3p expression by RT-qPCR during HSC activation. All results of relative expression values are shown as the mean ± SEM. of triplicate experiments. The mRNA expression was examined by RT-qPCR analysis and normalized to *GAPDH*. MiR-199a-3p expression was examined by RT-qPCR analysis and normalized to U6 expression. Statistical significances were analysed by one-way analysis of variance followed by a post-hoc LSD test, *P < 0.05 and **P < 0.01

To identify aberrant miRNAs during HSC activation, we firstly isolated rat primary HSCs from healthy livers. The freshly isolated primary HSCs were circular with lipid droplet and their morphologies were distinctly changed to be stellate during culture in vitro, suggesting their activation (Fig. 1a). The enhanced expression of fibrotic markers including FN, α-SMA, p-SMAD2/3 and CTGF further confirmed the activation of HSCs (Fig. 1b, c). Then, we assessed the miRNA expression profiles in quiescent and activated HSCs by miRNA microarray. The significant alteration of miRNA expression has been described in our previous reports.^15^ Among these dysregulated miRNAs, miR-199a-3p was significantly upregulated during HSC activation, and its increased expression was further validated by RT-qPCR analysis (Fig. 1d). These results indicate that miR-199a-3p may play a role during HSC activation.

### Increased miR-199a-3p expression in multiple liver injury models

Given the increased expression of miR-199a-3p in HSC activation and that HSCs undergo activation in response to liver injury,^22^ we assessed the level of miR-199a-3p in several liver injury models.

**Fig. 2:**
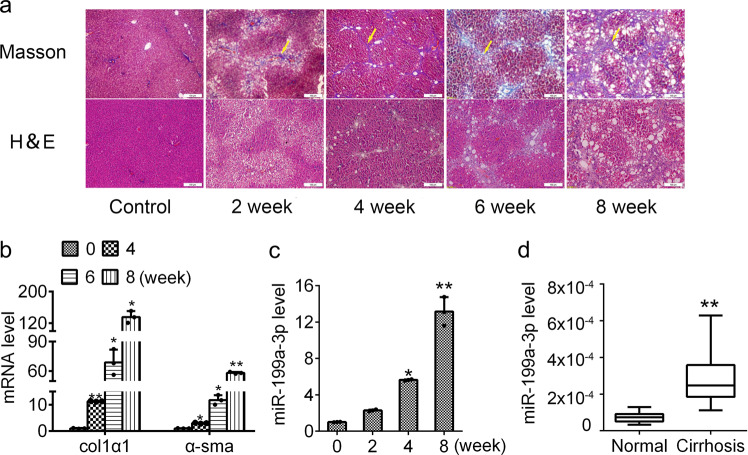
Increased miR-199a-3p expression in fibrotic liver tissues from rat, mouse and human. **a** Representative images (×100 magnification) of H&E-stained and Masson’s trichrome-stained liver sections of rat induced by CCl_4_ treatment for 2, 4, 6 or 8 weeks (*n* = 6 per group). The yellow arrow indicates fibrosis or fibrotic nodules. **b** The mRNA levels of *α-SMA* and *COL1α1* and **c** miR-199a-3p expression was measured at different stages of rat liver fibrosis (*n* = 6 per group). **d** miR-199a-3p level was determined in the livers of healthy and cirrhosis patients (*n* = 11 per group). The mRNA expression was examined by RT-qPCR analysis and normalized to *GAPDH* expression. MiR-199a-3p expression was examined by RT-qPCR analysis and normalized to U6 expression. Data (means ± SEM) are obtained from triplicate experiments (unpaired two-sample Student’s *t* test, *P < 0.05 and **P < 0.01)

Firstly, we used the well-established rodent model of carbon tetrachloride (CCl_4_)-induced liver fibrosis. To this end, we exposed rats to CCl_4_ subcutaneous injection twice a week and rats were injected with olive oil as control. As shown by haematoxylin and eosin (H&E) and Masson’s trichrome staining, the livers exhibited extensive steatosis and necrosis with inflammatory infiltrate but mild fibrosis after 2 weeks’ treatment with CCl_4_ (Fig. 2a). As the treatment proceeded, the livers gradually displayed advanced fibrosis with occurrence of spreading bridging fibrosis and fibrotic nodules (Fig. 2a). Besides, the expression of fibrotic marker genes (*α-SMA* and *COL1α1*) was significantly increased in the livers with fibrosis (Fig. 2b). These results support the successful establishment of liver fibrosis in rats. As shown in Fig. 2c, in parallel with the severity of liver fibrosis, miR-199a-3p hepatic expression gradually increased. Besides, we also observed greatly enhanced expression of miR-199a-3p in the cirrhotic liver samples from patients (Fig. 2d).

### miR-199a-3p promotes the expression of fibrotic markers in HSCs

**Fig. 3:**
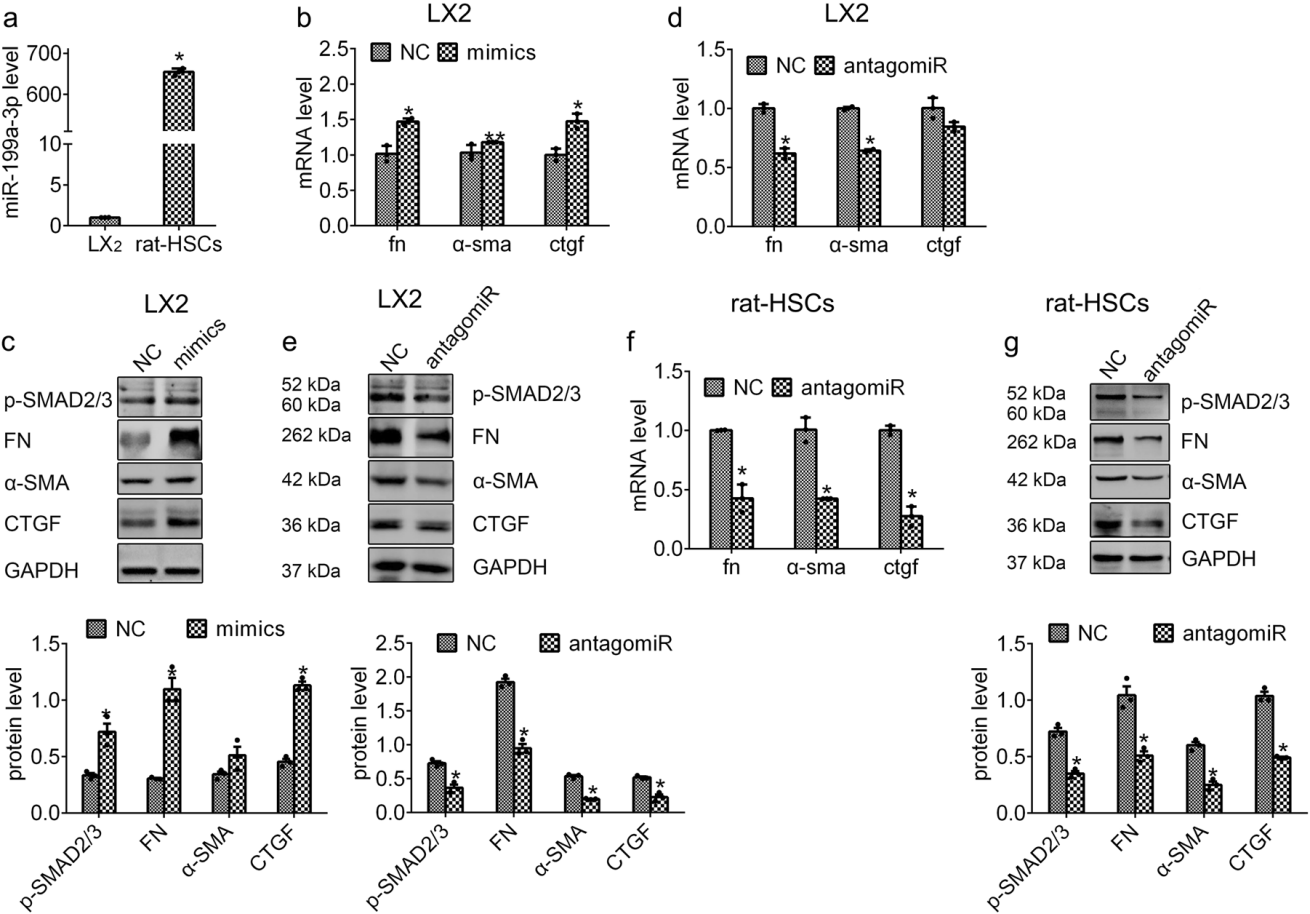
miR-199a-3p promotes HSC activation by enhancing the expression of fibrotic markers. **a** The level of miR-199a-3p in rat primary activated HSCs and human LX2 cells. **b** LX2 cells were transfected with miR-199a-3p mimics or NC-miR for 48 h. The mRNA level of *FN, α-SMA* and *CTGF* was detected by RT-qPCR and **c** the protein levels of FN, p-SMAD2/3, α-SMA and CTGF were examined by western blotting, respectively. LX2 cells were transfected with antagomiR-199a-3p or inhibitor NC for 48 h, and the expression of fibrotic markers were examined at mRNA (**d**) and protein levels (**e**), respectively. Rat HSCs were treated with antagomiR-199a-3p or inhibitor NC for 48 h, and the expression of these fibrotic markers expression were examined at mRNA (**f**) and protein levels (**g**), respectively. The mRNA expression was examined by RT-qPCR analysis and normalized to *GAPDH* expression. MiR-199a-3p expression was examined by RT-qPCR analysis and normalized to U6 expression. The relative value of protein band density was measured with Image J software and normalized to GAPDH. Data (means ± SEM) are obtained from triplicate experiments (unpaired two-sample Student’s *t* test, *P < 0.05 and **P < 0.01)

To investigate the effect of miR-199a-3p on HSC activation, we performed antagomiR-based silencing and mimics-induced overexpression of miR-199a-3p in HSCs, respectively. Firstly, we detected the endogenous miR-199a-3p expression in rat HSCs and human LX2 cells, and we found that activated rat HSCs expressed much higher miR-199a-3p than LX2 cells (Fig. 3a). Therefore, we conducted overexpression and knockdown experiments in LX2 cells, and knockdown experiments in activated rat HSCs. As expected, miR-199a-3p expression was obviously increased following miR-199a-3p mimics transfection in LX2 cells and significantly decreased after antagomiR treatment in activated rat HSCs (Supplementary Fig. 1C). After forced expression of miR-199a-3p, the expression of fibrotic markers (FN, α-SMA, CTGF) were greatly induced at both mRNA and protein levels (Fig. 3b, c). By contrast, transfection of antagomiR-199a-3p led to a significant decrease of fibrotic markers at both mRNA and protein level (Fig. 3d–g). Therefore, these data strongly support the notion that miR-199a-3p is an important mediator during HSC activation.

### miR-199a-3p regulates CAV2 expression

**Fig. 4:**
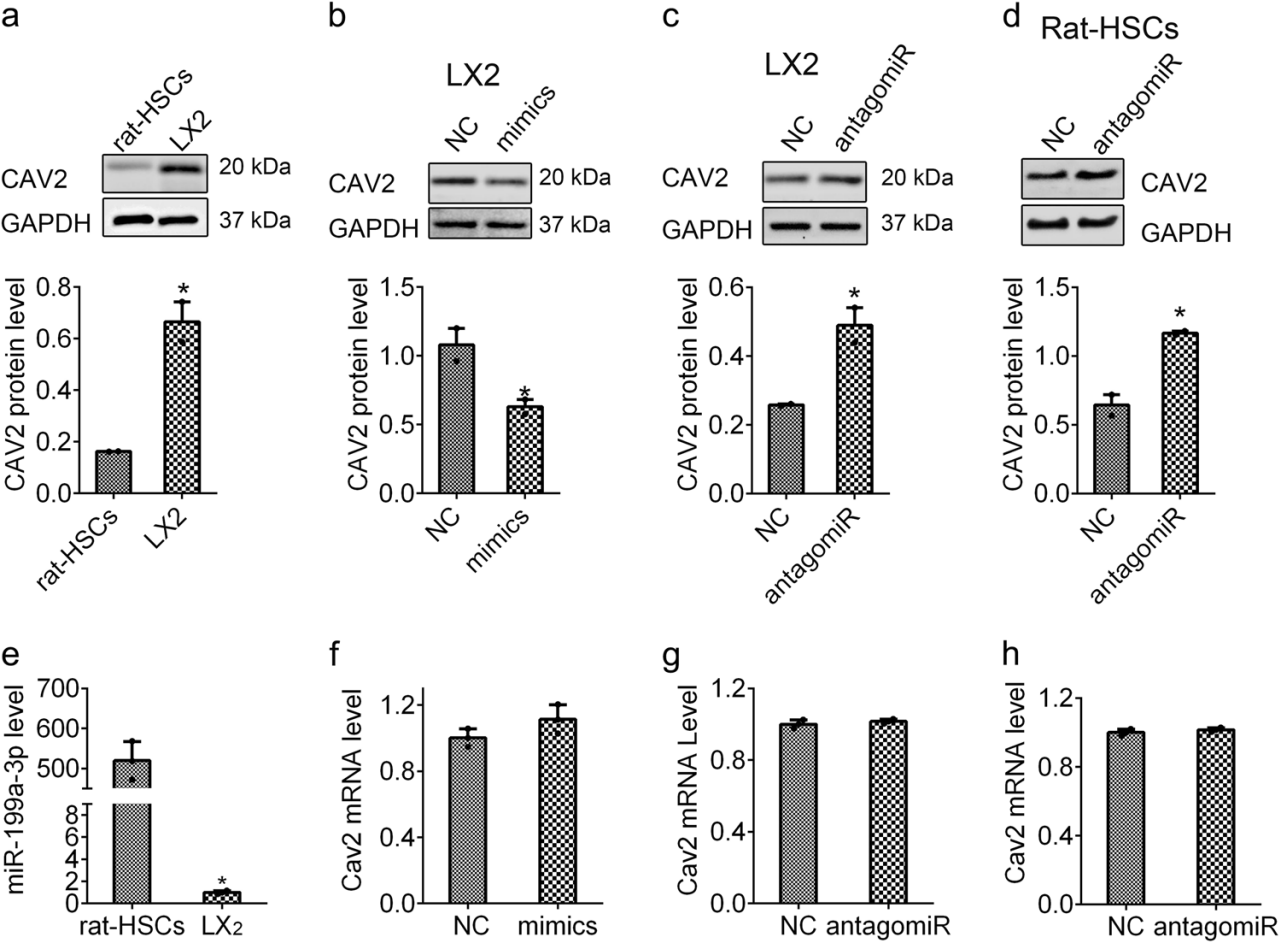
miR-199a-3p regulates CAV2 expression in HSCs. **a** CAV2 protein expression in rat activated HSCs and human LX2 cells. The protein levels of CAV2 after transfection of miR-199a-3p mimics in LX2 cells (**b**) or antagomiR in LX2 cells (**c**) and rat HSCs (**d**). **e** The miR-199a-3p level in rat activated HSCs and human LX2 cells. The mRNA levels of CAV2 after transfection of miR-199a-3p mimics in LX2 cells (**f**) or antagomiR in LX2 cells (**g**) and rat HSCs (**h**). The mRNA expression was examined by RT-qPCR analysis and normalized to *GAPDH* expression. The relative value of protein band density was measured with Image J software and normalized to GAPDH. Data (means ± SEM) are obtained from triplicate experiments (unpaired two-sample Student’s *t* test, *P < 0.05)

CAV2 has been demonstrated as a direct target of miR-199a-3p in breast cancer cells.^23^ And the prediction of bioinformatics (TargetScan, miRbase and miRanda) indicated that CAV2 may be the target of miR-199a-3p. However, the relevance between CAV2 and miR-199a-3p has not been studied in HSCs. We firstly analysed the endogenous expression of CAV2 and miR-199a-3p in rat HSCs and LX2 cells. Interestingly, we found that the higher the expression of miR-199a-3p, the lower the level of CAV2 protein in both cells, indicating there is a negative correlation between miR-199a-3p and CAV2 expression (Fig. 4a, e). We further examined the effect of miR-199a-3p on CAV2 expression by overexpression or knockdown strategies. As shown in Fig. 4b, f, miR-199a-3p overexpression reduced CAV2 protein expression in LX2 cells, but has negligible effect on *CAV2* mRNA level. By contrast, miR-199a-3p knockdown increased CAV2 protein level without effect on *CAV2* mRNA expression in both human LX2 and rat HSCs (Fig. 4c, d, g, h). So, these results indicate that miR-199a-3p negatively regulates CAV2 expression through repressing protein translation.

### CAV2 negatively mediated HSC activation through repressing TGFβRI expression

**Fig. 5:**
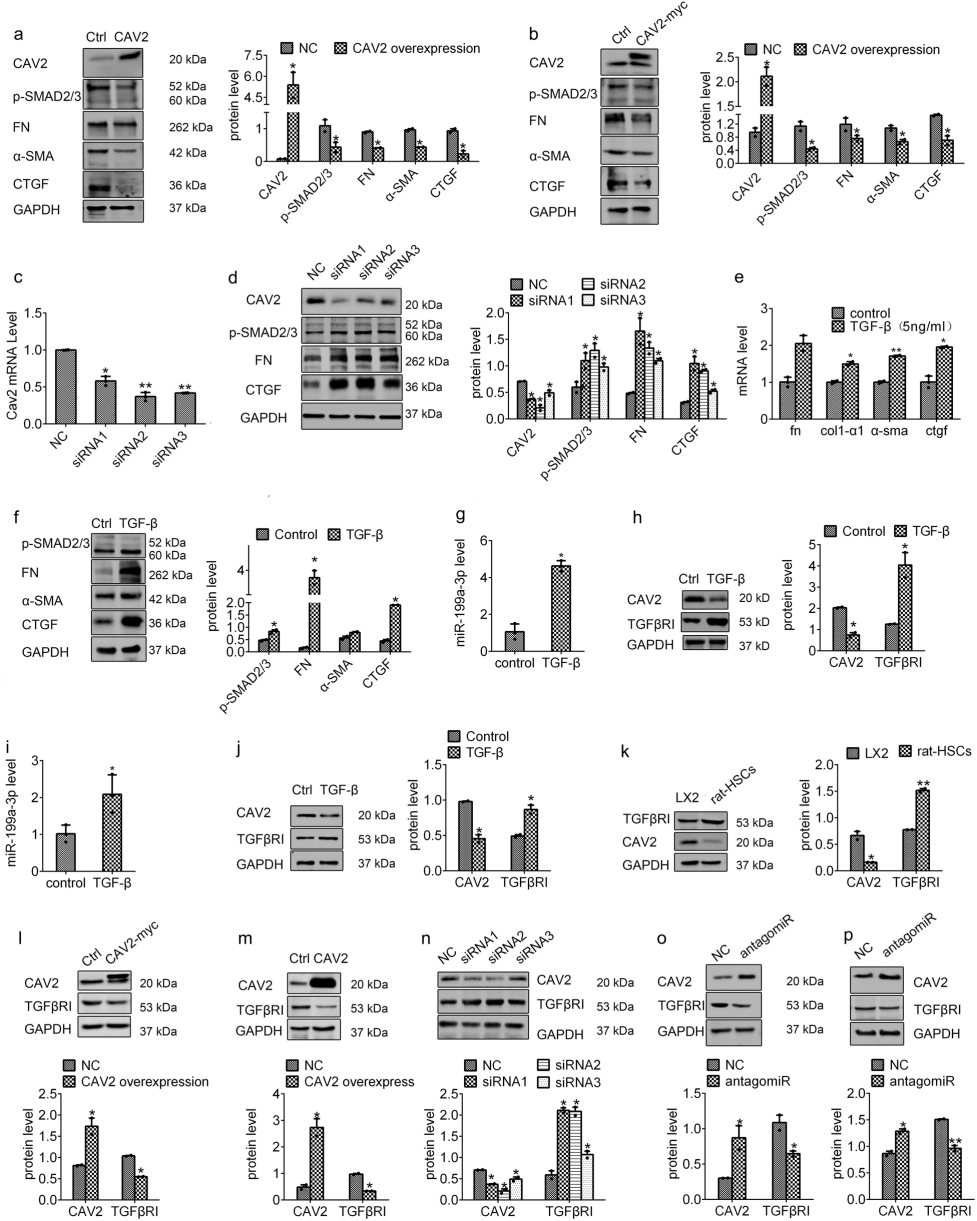
CAV2 inhibits HSC activation via TGFβRI repression. **a**, **b** Effects of CAV2 overexpression on the protein levels of CAV2, p-SMAD2/3, FN, α-SMA and CTGF in activated rat HSCs and human LX2 cells. **c**, **d** Effects of CAV2 knockdown by siRNAs in LX2 cells on the protein levels of CAV2, p-SMAD2/3, FN and CTGF. **e**, **f** The mRNA and protein levels of FN, COLIα1, α-SMA, CTGF and p-SMAD2/3 were measured after treatment with TGF-β for 24 h in LX2 cells. **g**, **h** LX2 cells and **i**, **j** activated rat HSCs were treated with TGF-β for 24 h, the expression of miR-199a-3p, CAV2 and TGFβRI was detected. **k** The protein expression of CAV2 and TGFβRI in activated rat HSCs and LX2 cells. Effects of CAV2 overexpression on TGFβRI protein level in LX2 cells (**l**) and activated rat HSCs (**m**). **n** Effect of CAV2 knockdown on TGFβRI protein level in LX2 cells. Effects of miR-199a-3p knockdown on the protein levels of CAV2 and TGFβRI in LX2 (**o**) and rat HSCs (**p**). The mRNA expression was examined by RT-qPCR analysis and normalized to *GAPDH* expression. MiR-199a-3p expression was examined by RT-qPCR analysis and normalized to U6 expression. The relative value of protein band density was measured with Image J software and normalized to GAPDH. Data (means ± SEM) are obtained from triplicate experiments (unpaired two-sample Student’s *t* test, *P < 0.05 and **P < 0.01)

To clarify how miR-199a-3p involves in liver fibrosis by regulating CAV2, we attempted to define CAV2 function in HSC activation and liver fibrosis. It has been reported that CAV2 deficiency increased fibrosis within Lewis lung carcinoma implanted into mice.^24^ However, the role of CAV2 in liver fibrosis remains obscure. Considering the low level of CAV2 in rat HSCs (Fig. 4a), we ectopically expressed CAV2 in rat HSCs to examine its effect on fibrosis. As shown in Fig. 5a, CAV2 overexpression strongly reduced the expression of fibrotic markers, such as CTGF, α-SMA, FN and p-SMAD2/3. Similar results were also observed in LX2 cells (Fig. 5b). Moreover, CAV2 knockdown by siRNAs dramatically increased the expression of fibrotic markers (Fig. 5c, d). Thus, these results indicate that CAV2 plays an anti-fibrotic role during HSC activation.

TGF-β acts as a pro-fibrogenic factor stimulating HSC activation and liver fibrosis.^25^ In TGF-β signalling pathway, TGF-β binds to a heterodimer of TGF-β type II and TGFβRI receptors, and activates the phosphorylation of the downstream Smad proteins, which are subsequently imported into the nucleus to regulate downstream gene expression.^26^ As analysed by RT-qPCR and western blotting assays, the expression of fibrotic markers increased after TGF-β treatment in LX2 cells, confirming the effective treatment of TGF-β on HSCs (Fig. 5e, f). Next, we examined the effect of TGF-β on miR-199a-3p and CAV2 expression, and found that TGF-β treatment indeed increased miR-199a-3p expression and decreased CAV2 expression in LX2 cells (Fig. 5g, h), further supporting the negative regulation of CAV2 by miR-199a-3p. Given the participation of caveolins in TGFβ receptors degradation,^27^ we also examined the TGFβRI expression and found the upregulation of TGFβRI in response to TGF-β treatment (Fig. 5h). Moreover, similar results were also observed in rat HSCs (Fig. 5i, j), suggesting the negative correlation between TGFβRI and CAV2.

To gain further insights into the association between CAV2 and TGFβRI, we firstly assessed the expression status of these two genes in LX2 cell and rat HSCs. As shown in Fig. 5k, CAV2 protein level was negatively correlated with TGFβRI level in these two cells. To further test whether CAV2 affects TGFβRI expression, we overexpressed CAV2 in both cells and indeed observed the reduced TGFβRI level (Fig. 5l, m). Moreover, CAV2 knockdown by siRNAs led to a strong increase in TGFβRI level in LX2 cells (Fig. 5n). These results indicate that CAV2 negatively regulates TGFβRI expression, thus suppressing TGF-β pathway. To demonstrate the involvement of miR-199a-3p in the TGF-β pathway, we inhibited miR-199a-3p expression by antagmiRs in rat HSCs and human LX2 cells. As expected, antagomiR-199a-3p treatment led to increased CAV2 expression, which in turn decreased TGFβRI protein expression in both cells (Fig. 5o, p). Besides, as shown in Fig. 3c, e, g, miR-199a-3p overexpression increased p-SMAD2/3 level, a hallmark of the TGF-β pathway, whereas inhibition of miR-199a-3p reduced its level. Taken together, our data provide strong evidences that miR-199a-3p activates TGF-β signalling pathway, probably by regulating CAV2-dependent TGFβRI, thus to stimulate HSC activation.

### MiR-199a-3p expression is controlled by Twist1 in HSCs

**Fig. 6:**
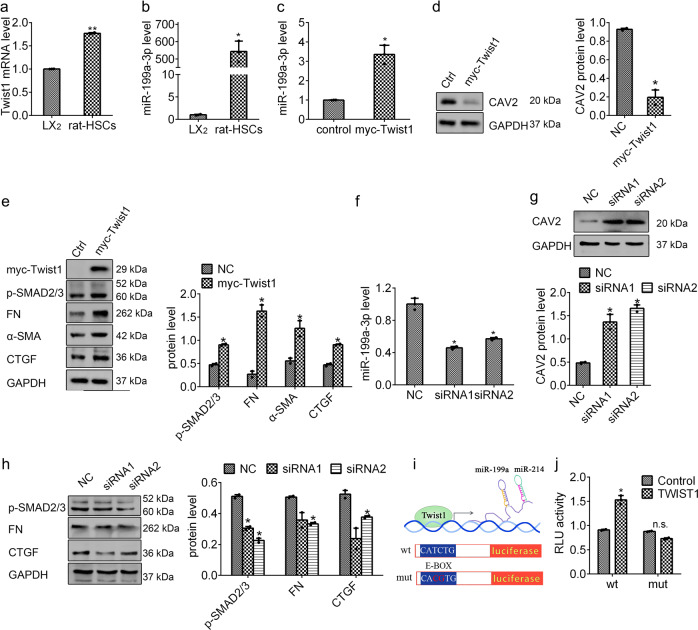
Twist1 drives miR-199a-3p expression and promotes HSC activation. **a**, **b** The expression of *Twist1* and miR-199a-3p in rat HSCs and LX2 cells. Effects of myc-tagged Twist1 on the expression of miR-199a-3p (**c**), CAV2 protein (**d**) and p-SMAD2/3, FN, α-SMA and CTGF (**e**) in LX2 cells. Effects of Twist1 knockdown in rat HSCs on the expression of miR-199a-3p (**f**), CAV2 protein (**g**), and p-SMAD2/3, FN and CTGF (**h**). **i** Schematic construction of luciferase reporter constructs harbouring wild-type or mutated miR-199a-3p promoter region. **j** Effects of Twsit1 expression on the relative luciferase activity (RLU). The mRNA expression was examined by RT-qPCR analysis and normalized to *GAPDH* expression. MiR-199a-3p expression was examined by RT-qPCR analysis and normalized to U6 expression. The relative value of protein band density was measured with Image J software and normalized to GAPDH. Data (means ± SEM) are obtained from triplicate experiments (unpaired two-sample Student’s *t* test, *P < 0.05 and **P < 0.01). n.s. non-significant

As a helix-loop-helix transcription factor, Twist1 has been defined to drive miR-199a/214 cluster expression by an E-Box promoter element during development.^28^ Our previous study has proved that Twist1 promotes miR-214 expression in HSCs.^15^ Considering that miR-199a-3p and miR-214 are transcribed from the same miR-199a/214 cluster, we attempted to clarify the functionality of Twist1 on miR-199a-3p in HSCs. To this end, we firstly examined the expression of *Twist1* in rat HSCs and LX2 cells and showed a positive correlation between *Twist1* and miR-199a-3p expressions (Fig. 6a, b). Then we successfully expressed Twist1 in LX2 cells (Fig. 6e and Supplementary Fig. 2A) and detected a remarkable increase of miR-199a-3p level and decrease of CAV2 protein (Fig. 6c, d). Moreover, Twist1 knockdown in rat HSCs resulted in an evident reduction of miR-199a-3p and upregulation of CAV2 protein (Supplementary Fig. 2B and Fig. 6f, g). These data reveal that Twist1 promotes miR-199a-3p expression and suppresses CAV2 protein in HSCs. Taking together the findings that both miR-199a-3p and miR-214 induced by Twist1 are pro-fibrotic molecules,^15^ we hypothesised that Twist1 functions as a pro-fibrotic molecule in HSCs. As shown in Fig. 6e, h, Twist1 overexpression in LX2 cells led to an increase of fibrotic markers, while Twist1 knockdown in rat HSCs resulted in a reduction of fibrotic markers. So, these results demonstrate that Twist1 can induce miR-199a-3p expression, which represses CAV2 protein and subsequently promotes HSC activation.

To further investigate whether Twist1 directly binds to the E-Box of miR-199a-3p promoter to drive its expression in HSCs, we amplified the E-box region and cloned to the luciferase reporter vector. In addition, the mutant construct (CATCTG mutated to CACGTG) was also generated as control to destroy TWIST1 potential binding site (Fig. 6i). As shown in Fig. 6j, Twist1 overexpression increased the luciferase activity in the presence of the wild-type E-box construct only, supporting that Twist1 directly binding to the E-box of miR-199a-3p gene promoter to trigger its expression. Taken together, our results indicate that Twist1 serves as a pro-fibrotic element involved in HSC activation by inducing miR-199a-3p expression.

### AntagomiR-199a-3p ameliorates liver fibrosis induced by CCl_4_ in mice

**Fig. 7:**
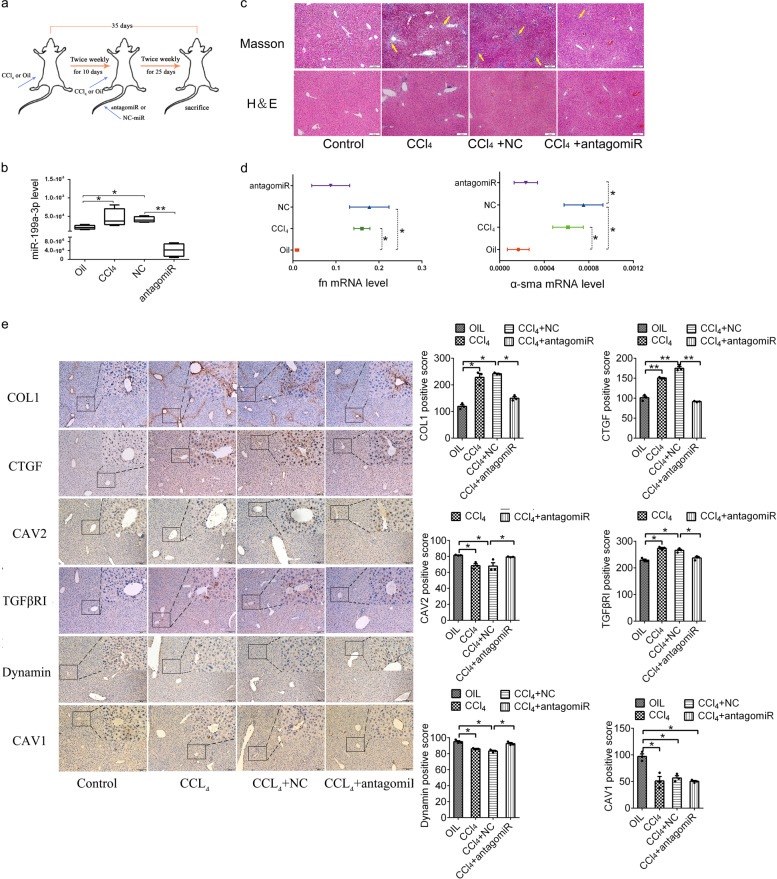
AntagomiR-199a-3p ameliorates CCl_4_-induced liver fibrosis in mice. **a** Schema of the injections in mice liver fibrosis model. **b** miR-199a-3p levels in livers from olive oil-treated, CCl_4_-treated, CCl_4_/NC-treated, CCl_4_/antagomir-199a-3p-treated mice (*n* = 5 per group). **c** H&E and Masson’s trichrome staining of liver sections from each group mice at ×100 magnification (*n* = 5 per group). The yellow arrow indicates fibrosis of livers. **d** The mRNA level of *FN, α-SMA* in each group mice livers (*n* = 5 per group). The yellow arrow indicates fibrosis of livers. **e** The immunohistology of COLI, CTGF, CAV2, TGFβRI, dynamin and CAV1 protein in each group mice livers (*n* = 5 per group). The mRNA expression was examined by RT-qPCR analysis and normalized to *GAPDH* expression. MiR-199a-3p expression was examined by RT-qPCR analysis and normalized to U6 expression. Data (means ± SEM) are obtained from triplicate experiments (one-way analysis of variance followed by a post-hoc LSD, *P < 0.05 and **P < 0.01)

To evaluate whether inhibiting miR-199a-3p could serve as an approach to treat liver fibrosis in vivo, miR-199a-3p antagomiR was intravenously injected into mice during liver fibrogenesis. Mice were subjected to CCl_4_ or olive oil twice a week for 5 weeks, followed by intravenous injection of antagomiR-199a-3p or scramble (NC)-miR twice a week via tail vein starting at the 10th day after CCl_4_ injection (Fig. 7a). As expected, miR-199-3p liver expression was induced during CCl_4_ treatment, and antagomiR-199a-3p efficiently dramatically inhibited miR-199-3p expression in mice livers (Fig. 7b). Then the extent of liver fibrosis was evaluated by Masson’s trichrome and H&E staining. As shown in Fig. 7c, the pronounced liver fibrosis was detected in CCl_4_-treated group. Intriguingly, antagomiR-199a-3p injection significantly ameliorated liver fibrosis of CCl_4_-treated mice when compared with NC-miR injection. Meanwhile, the expression of fibrotic markers in livers was greatly enhanced after CCl_4_ treatment and obviously reversed by subsequent antagomiR-199-3p injection (Fig. 7d). Besides, we observed CAV2 protein expression was reduced after CCl_4_ treatment and recovered by subsequent antagomiR-199-3p injection, and the corresponding changes of TGFβRI expression in the opposite way, accompanied by decreased dynamin in CCl_4_-treated group and increased dynamin in antagomiR-199a-3p group, indicating the inhibited endocytosis in the CCl_4_-treated group and active endocytosis in antagomiR-199a-3p group. And CAV1 expression was reduced in the CCl_4_-treated group, consistent with previous reports,^29^ but not reversed by subsequent antagomiR-199-3p injection. Taken together, these results indicate that silencing miR-199a-3p attenuates CCl_4_-induced liver fibrosis in mice, implying the potential of miR-199a-3p as a target for the treatment of hepatic fibrosis in vivo.

## Discussion

Liver fibrosis is caused by various chronic liver diseases, and eventually proceeds to cirrhosis and even HCC. Up to 90% of HCC develops on the basis of liver fibrosis or cirrhosis.^30^ It is widely accepted that HSC activation is a key event in liver fibrogenesis. Increasing evidences have demonstrated that HSC activation contributes 85–95% of the hepatic myofibroblasts in fibrosis triggered by NAFLD, hepatotoxins or biliary injury.^31^ Therefore, investigating the mechanism underlying HSC activation may provide new therapeutic opportunities for liver fibrosis and cirrhosis.

Consistent with previous reports, miR-378 and miR-29a were downregulated, while miR-19b, miR-221 were upregulated during HSC activation in our data of microRNA microarray.^14,19,21^ We also detected both enhanced miR-199a-5p and miR-199a-3p expression in our array data, which share the same precursor, and miR-199a-5p has been proved to participate in lung fibrosis, oral submucous fibrosis and peritoneal fibrosis, and HSC activation.^32–34^ So, we examined the level of miR-199a-3p and miR-199a-5p in quiescent and activated HSCs and found a much higher level of miR-199a-3p (Supplementary Fig. 1A), yielding that miR-199a-3p is a major mature product expressed in HSCs, in accordance with the miRbase data. Therefore, miR-199a-3p was identified as the candidate microRNA for further analysis. The upregulation of miR-199a-3p in activated HSCs and fibrotic liver tissues of rat, mouse and human had been reported previously.^19,35–37^ However, its precise function and mechanism in liver fibrosis remain poorly understood. Concordant with previous findings, our results showed an enhanced expression of miR-199a-3p in activated HSCs, and fibrotic liver tissues from rat and human (Figs. 1 and 2). Given that the major cell type in the liver is hepatocytes, and HSCs constitute only <1% of the total cell population,^38^ we detected miR-199a-3p level in activated HSCs and hepatocytes from rat, and found a strikingly higher level in activated HSCs than in hepatocytes (Supplementary Fig. 1B). Moreover, we demonstrated that an increased level of miR-199a-3p in TGF-β-treated HSCs, while miR-199a-3p level in TGF-β-treated hepatocytes did not change (Supplementary Fig. 1B). These results suggest that miR-199a-3p exerts major and specific functions in HSCs. Then, we confirmed the promotive effects of miR-199a-3p on HSC activation by enhanced the expression of fibrotic markers. However, in rat activated HSCs with miR-199a-3p mimics transfection, only the protein of p-SMAD2/3, CTGF slightly increased with little changes of FN and α-SMA (Supplementary Fig. 1D), possibly because the sufficiently activated rat-HSCs we adopted actually express an extremely high level of miR-199a-3p and fibrotic markers, and it could not be further induced, reflecting the fact that the regulation of miR-199a-3p is not unlimited, and the possible existence of some negative feedback loops to suppress the function of miR-199a-3p in liver fibrosis. To explore the clinical implications, we knocked down miR-199a-3p expression in vivo experiment, and consequently, it reduced the expression of fibrotic markers, and led to the remission of liver fibrosis, supporting the role of miR-199a-3p in treating liver fibrosis.

MiR-199a gene is located in a cluster with miR-214 from the DNM3 introns.^39^ E-box element exists in the promoter region of the miR-199a/miR214 cluster and is responsive to the transcription factor Twist1. In epithelial ovarian cancer stem cells, Twist1 has been identified as the positive regulator of this gene cluster.^40^ However, in other tumour cell lines, Twist1 has no effect on miR-199a-3p expression.^41^ In this study, we found that Twist1 can induce miR-199a-3p expression in HSCs and further promote HSC activation (Fig. 6). Besides, Twist1 protein level was upregulated in response to TGF-β treatment in LX2 cells, suggesting that TGF-β may modulate miR-199a-3p expression, partially through elevated Twist1 expression (Supplementary Fig. 2C). Interestingly, higher Twist1 expression has been observed previously in patients with liver cirrhosis and HCC.^15,42^ In HCC, Twist1 mediates tumour invasion and metastasis by regulating epithelial to mesenchymal transition and its expression can be induced by EGF and TGF-β.^43^ In view of Twist1 being implicated in both liver fibrosis and HCC, investigation of Twist1 inhibitors to prevent the development of liver fibrosis and HCC is warranted for further study in pre-clinical animal models. Since this study and our previous report^15^ have proved that Twist1 drives the expression of both miR-199a-3p and miR-214 which promote liver fibrosis through different mechanisms, whether or not this indicates the inhibition of Twist1 is more effective in treating liver fibrosis than inhibiting miR-199a-3p or miR-214 alone?

We dissected the molecular mechanism by which increased miR-199a-3p promotes liver fibrosis. Shatseva et al. showed that CAV2 could be a target for miR-199a-3p in rat.^23^ Bioinformatics have predicted several genes related to fibrosis are potential targets of miR-199a-3p, such as PTEN, CDK7 and CAV2. We have detected the protein levels of PTEN, CDK7 and CAV2 in LX2 cells after miR-199a-3p mimics or antagomir-199a-3p transfection. And we discovered there is no obvious corresponding change in the protein levels of PTEN and CDK7 (data not shown), while CAV2 protein level was significantly altered (Fig. 4). Accordingly, we speculated CAV2 is implicated in miR-199a-3p-induced liver fibrosis, and explored the further mechanism. Considering the 3′UTR of human and rat CAV2 mRNAs are different, we cloned human CAV2 3′UTR containing the predicted sites of miR-199a-3p to the luciferase reporter vector. However, the luciferase activity was unchanged by miR-199a-3p mimics treatment, indicating that there are no functional target sites in 3′UTR of CAV2 in human (data not shown). We speculated that the target sites of microRNAs might be outside of 3′UTR,^44,45^ and then we divided the full length of human CAV2 mRNA into four fragments and cloned them into a luciferase reporter vector separately. However, there are still no changes observed in luciferase assay (data not shown), revealing that miR-199a-3p may regulate CAV2 expression through other possible pathways. Based on the fact that CAV1 is essential for CAV2 functions, we sought to determine whether CAV1 mediated the regulation of miR-199a-3p on CAV2. To address this question, we examined CAV1 expression in HSCs after transfection of miR-199a-3p mimics or antagomiR. The data showed that miR-199a-3p overexpression or knockdown has minimal effect on CAV1 expression (Supplementary Fig. 1e, f). Besides, knockdown of miR-199a-3p expression in vivo experiment did not affect CAV1 expression in liver tissues (Fig. 7e). Taken together, miR-199a-3p is probably to regulate CAV2 expression indirectly and independently of CAV1 in HSCs. However, the exact regulatory mechanism remains to be unearthed.

Up to date, it is largely unknown how CAV2 is implicated in fibrosis. Liu Y et al. found increased fibrosis in CAV2-KO mice following Lewis lung carcinoma tumours implantation, while Yokomori H et al. reported the protein expression of CAV2 significantly increased in cirrhotic liver tissues compared with normal liver.^24,46^ Of note, in the study of Yokomori H et al., they used the cirrhotic liver tissues from patients with primary biliary cirrhosis, whose bile ducts are badly damaged, contributing to high expression of CAV2. Here, we identified that CAV2 suppresses HSC activation and its anti-fibrotic role in liver fibrosis (Fig. 5a–d). Although our data discovered the function of CAV2 in HSCs, it may also have a role in biliary epithelial cells. In addition, we unfolded that CAV2 negatively regulates the TGF-β pathway by reducing TGFβRI expression to suppress HSC activation. TGF-β receptors are supposed to be modulated negatively by caveolins through promoting its degradation.^27^ The prevailing view is that caveolins regulate TGFβRI degradation probably via Smad7, which recruits E3 ubiquitin ligases Smurf1/2 and induces ubiquitination, followed by degradation depending on proteasome and lysosome.^47^ The interaction of CAV1 and TGFβRI has been reported in NIH-3T3 cells.^48^ In this study, we elucidated the effect of CAV2 on TGFβRI. CAV2 overexpression or knockdown obviously altered TGFβRI protein expression in HSCs (Fig. 5l–n). Moreover, antagomiR-199a-3p transfection resulted in reduced TGFβRI protein concurrent with increased CAV2 protein, suggesting miR-199a-3p may affect TGFβRI expression through CAV2 inhibition (Fig. 5o, p). Besides, antagomiR-199a-3p injection in vivo experiment also revealed that inhibition of miR-199a-3p-induced CAV2 protein and reduced TGFβRI protein, accompanied by increased dynamin protein, indicating the increased endocytosis. On the other hand, we found miR-199a-3p and CAV2 are regulated by TGF-β in HSCs (Fig. 5g–j). Interestingly, it is informative that TGF-β-mediated miR-199a-3p upregulation and CAV2 reduction may promote the TGF-β signalling pathway by increasing TGFβRI in a positive feedback loop in vivo.

## Conclusions

**Fig. 8:**
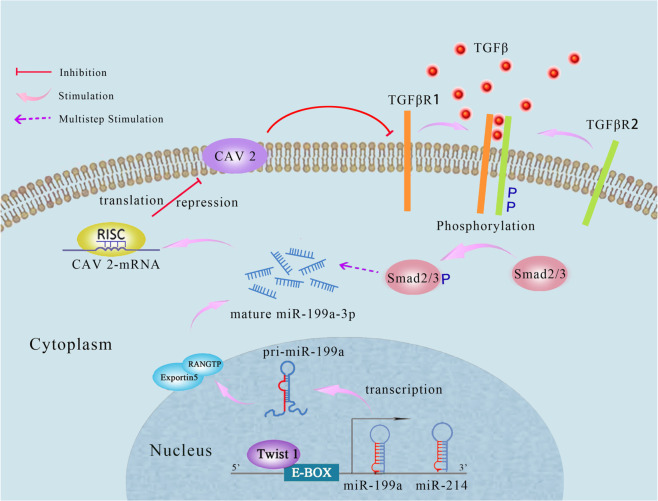
Proposed model for the involvement of miR-199a-3p and CAV2 in hepatic fibrosis. miR-199a-3p expression is induced by Twist1 via binding with the E-Box element of miR-199a-3p gene promoter and TGF-β treatment in HSCs. Consequently, increased miR-199a-3p inhibits CAV2 expression, which upregulates TGFβRI level and subsequently accelerates TGF-β signalling pathway, thereby promoting HSC activation

In this study, we proposed a novel mechanism for miR-199a-3p involvement in liver fibrosis. When HSCs are exposed to the stimulus TGF-β, Twist1 expression is induced and then Twist1 is exported to the nucleus and binds with the E-box element of miR-199a-3p gene to trigger its expression. Subsequently, miR-199a-3p represses CAV2 protein. As a consequence, CAV2-mediated TGFβRI degradation is inhibited, thus promoting TGF-β pathway. The Smad2/3 as a downstream substrate is phosphorylated and further promotes the expression of pro-fibrotic genes and HSC activation. Therefore, our study indicates the presence of the positive feedback loop between miR-199a-3p and TGF-β signalling pathway, which represents a novel mechanism involved in HSC activation (Fig. 8). Importantly, silencing of miR-199a-3p by antagomiR attenuates CCl_4_-induced liver fibrosis in mice, demonstrating that miR-199a-3p could be an attractive therapeutic target for liver fibrosis.

## Materials and methods

### Isolation of rat HSCs and cell culture

Normal male Sprague-Dawley rats (Animal Centre of Sichuan university, weighting 300–400 g) were used for primary HSCs isolation. All animal studies were approved by the Medical Ethics Committee of the West China Hospital of Sichuan University. Rat primary HSCs were isolated as previously described and the cell purity was more than 95%.^49^ The rat primary HSCs were grown in low glucose Dulbecco’s modified Eagle medium (DMEM) (Gibco, USA) with 20% fetal bovine serum (FBS) (Biological Industries, Israel). Culture medium was replaced every 48 h and cells were incubated at 37 °C with 5% CO_2_.

Immortalized human HSC cell line LX2 (Procell, Wuhan, China) was cultured in high glucose DMEM (Gibco, USA) with 10% FBS and maintained in 37 °C incubator with 5% CO_2_.

### Liver histology staining and Immunohistochemistry

Liver specimens from rat or mouse were fixed in 4% paraformaldehyde, embedded in paraffin, and cut into sections. The sections were then deparaffinized, hydrated and stained with hematoxylin–eosin (H&E) (Beyotime, Shanghai, China) and Trichrome (Masson) stain kit (Baso diagnostics, Zhuhai, China). Hepatic lipid content was quantified on fresh frozen sections using Oil Red O staining (Beyotime, Shanghai, China). For immunohistochemistry, the liver sections are incubated in 3% H_2_O_2_ for 25 min following antigen retrieval, and then blocked with 3%BSA for 30 min. After that, the sections were incubated with the following primary antibody at 4 °C overnight: mouse anti-CAV2 (ab2911; Abcam, Cambridge, UK), mouse anti-CAV1 (GB11409; Servicebio; Wuhan; China), mouse anti-collagen1α1 (GB11022-3; Servicebio; Wuhan; China), rabbit anti-CTGF (ab6992; Abcam, Cambridge, UK), rabbit anti-TGFβRI (sc-398; Santa Cruz Biotechnology, CA, USA), rabbit anti-α-Dynamin2 (ab151555; Abcam, Cambridge, UK), and then with a biotinylated secondary antibody for 50 min. The expression was visualized by 3,30-diaminobenzi-dine tetrahydrochloride (DAB) staining. And the immunohistochemistry is quantified by Image J software.

### Human liver tissues collections

Liver tissues were obtained from hospitalized patients with liver cirrhosis or other liver diseases in West China Hospital of Sichuan University. Cirrhotic liver tissues were selected and liver tissues without fibrosis (the remote tissues of liver hemangioma) were collected for this study. The tissues were instantly stored in liquid nitrogen when surgically resected. The informed consent was obtained from all patients, and the study was approved by the Human Ethics Committee of Sichuan University.

### Cell transfection and treatment

All miRNA mimics, antagomiRs, and siRNAs were chemically synthesized (GenePharma, Shanghai, China) and the sequences were as follows: 5′-GGCUCAACUCGCAUCUCAATT-3′ for human CAV2 siRNA1, 5′-CCCUCUUUGAAAUCAGCAATT-3′ for human CAV2 siRNA2, 5′-GAUGUUAUCAUUGCUCCAUTT-3′ for human CAV2 siRNA3, 5′-GGUACAUCGACUUCCUGUATT-3′ for rat TWIST1 siRNA1, 5′-UGGCAAGCUGCAGCUAUGUTT-3′ for rat TWIST1 siRNA2, 5′-ACAGUAGUCUGCACAUUGGUUA-3′ for miR-199a-3p mimics, and 5′-UUCUCCGAACGUGUCACGUTT-3′ for NC-miR, 5′-UAACCAAUGUGCAGACUACUGU-3′ for antagomiR-199a-3p mimics, and 5′-CAGUACUUUUGUGUAGUACAA-3′ for inhibitor NC. All the transfections were performed in 3rd–5th passage rat activated HSCs or human LX2 cells using RNAimax (Invitrogen, Carlsbad, CA, USA) at a final concentration of 100 nM for 48 h. Rat activated HSCs or human LX2 cells were transfected with plasmid using Lipofectamine 2000 (Invitrogen, Carlsbad, CA, USA) for 72 h. TGF-β1 (PeproTech, USA) treatment was performed in 3rd–5th passage rat activated HSCs or human LX2 cells at 5 ng/ml for 24 h following starved with FBS-free DMEM for 6 h. Total RNAs and proteins were collected for qRT-PCR and western blotting, respectively.

### qRT-PCR

Total RNA was extracted using TRIzol reagent as per the manufacturer’s instructions. For miRNA quantification, synthesis of cDNA was performed with Reverse Transcription Kit (GeneCopoeia, Rockville, MD, USA), and quantified using Hairpin-it^TM^ miRNA RT-PCR Quantitation Kit (GenePharma, Shanghai, China). For mRNA quantification, total RNA was reverse transcribed using High Capacity cDNA Reverse Transcription Kit (Applied Biosystems, Foster City, CA, USA), and target gene levels were detected with SYBR Green Mix (Life Technologies, Grand Island, NY, USA). The primers used are listed in Supplementary Table 1.

### Western blot analysis

Total protein was extracted from cells in RIPA lysis buffer (50 mM Tris-HCl, pH 7.4, 100 mM 2-Mercaptoethanol, 2% w/v SDS, 10% glycerol) with protease inhibitors and phosphatase inhibitors (Selleck, Boston, MA, USA). Protein concentration was measured by Pierce BCA Protein Assay Kit (Thermo Scientific, USA). After separation by 8% or 12% SDS PAGE, protein was transferred onto PVDF membrane (Millipore, Billerica, MA, USA). Primary antibodies used were as follows: mouse anti-CAV2 (610684; BD Biosciences, San Diego, CA, USA) used to detect human and rat CAV2, rabbit anti-CAV2 (D4A6; cell signalling Technologies, Danvers, MA, USA) used to measure human CAV2, rabbit anti-CAV1 (D46G3; cell signalling Technologies, Danvers, MA, USA), mouse anti-α-SMA (ab7817; Abcam, Cambridge, UK), rabbit anti-pSMAD2/3 (D27F4, cell signalling Technologies, Danvers, MA, USA), rabbit anti-FN (ab2413; Abcam, Cambridge, UK), rabbit anti-CTGF (ab6992; Abcam, Cambridge, UK), rabbit anti-TGFβRI (sc-398; Santa Cruz Biotechnology, CA, USA), mouse anti-twist1 (ab50887; Abcam, Cambridge, UK), mouse anti-myc tag (sc-40; Santa Cruz Biotechnology, CA, USA) and rabbit anti-GAPDH (#3683; cell signalling Technologies, Danvers, MA, USA), rabbit anti-β-actin (13E5; Cell Signalling Technologies, Danvers, MA, USA). Horseradish peroxidase conjugated anti-rabbit (Sigma, USA) or anti-mouse IgG (GE, USA) was used as secondary antibody. Protein bands were visualized by SuperSignal West Dura Extended Duration Substrate (Thermo Scientific, USA).

### Plasmid construction

The LGFP-CAV2 and LGFP-C-myc-CAV2 for CAV2 overexpression was constructed by Genewiz (Suzhou, China). The Twist1 sequence was amplified and cloned into the lentiviral vector pCDH-CMV-MCS-EF1-copGFP, constructing the overexpression plasmid. Supplementary Table 2 lists the primers for plasmid construction.

The 3′-UTR region of human CAV2 mRNA containing the potential miR-199a-3p binding site was cloned into pmirGLO Dual-Luciferase miRNA Target Expression Vector (Promega, Madison, WI, USA). The full length of human CAV2 mRNA was divided into four fragments and then these fragments were cloned into the luciferase reporter vector, respectively, constructing the pMir-Report-CAV2 reporter plasmids.

The miR-199a-3p promoter region was amplified from the rat HSCs genomic DNA, and cloned into pGL4.27 vector (Promega, Madison, WI, USA). The mutant miR-199a-3p promoter containing 2-base point mutation (CATCTG→CACGTG) in the E-box site was generated by using the QuikChange site-directed mutagenesis Kit (Aglient technologies) and verified by DNA sequencing.

### Luciferase reporter assay

For investigating whether Twist1 binds to the promoter of miR-199a-3p gene, we transfected HEK 293T cells with the recombinant pGL4.27 plasmid containing wild-type or mutant miR-199a-3p gene promoter and Twist1 overexpression plasmid or control plasmid, and pRL-TK containing renilla luciferase reporter gene as a control for transfection efficiency. After 24 h, we detected firefly and Renilla luciferase activities using the Dual-Luciferase Assays Kit (Promega, Madison, WI, USA). Renilla luciferase activity was used for normalization.

For examining whether miR-199a-3p directly target CAV2 mRNA, pMir-Report-CAV2 reporter plasmids containing the 3′-UTR or the fragments of CAV2 mRNA were transfected into HEK 293T cells in the presence of miR-199a-3p mimics or scramble using Lipofectamine 2000 for 24 h. Then, luciferase activities were measured.

### Experimental animals models

Male Sprague-Dawley rats (6–8 weeks, 200–300 g) and C57BL/6J mice (6–8 weeks, 20–22 g) were purchased from the Experimental Animal Centre of Sichuan University. All animal studies were approved by the Medical Ethics Committee of the West China Hospital of Sichuan University.

For CCl_4_-induced liver fibrosis model, rats were received 0.3 mL of olive oil (*n* = 20) or CCl_4_/olive oil (3:2, v/v) (*n* = 24) per 100 g body weight by subcutaneously injection twice a week for 2, 4, 6 and 8 weeks, and then rats were sacrificed to evaluate whether modelling is successful.

To examine the effect of anragomiR-199a-3p in vivo, C57BL/6J mice were randomly divided into four groups: Olive oil group, CCl_4_ group, CCl_4_/NC (Scramble-miR) group, CCl_4_/antagomiR-199a-3p group. Firstly, mice were received 0.1 mL of olive oil (*n* = 5) or CCl_4_/olive oil (1:3, v/v) (*n* = 15) per 100 g body weight by intraperitoneally injection twice a week. At the 10th day, each mice was treated with 62.5 nmol scramble-miR (*n* = 5) or antagomiR-199a-3p (*n* = 5) via tail vein injection or sham injection (*n* = 10) twice a week in parallel with olive oil or CCl_4_/olive oil injection. Mice were sacrificed at 5 weeks after first injection and the livers were collected for further analysis.

### Statistical analysis

These data are expressed as the mean ± standard deviation (SD) and are representative of at least three independent experiments. Statistical significance among groups were analysed by unpaired two-sample Student’s *t* test or one-way analysis of variance followed by a post-hoc LSD test. *P* < 0.05 was considered statistically significant. Statistical analyses were performed using IBM SPSS Statistics 21 software (Release version 21.0.0.0, IBM Corp, Armonk, NY, USA).

## Supplementary information

Twist1-induced miR-199a-3p promotes liver fibrosis by suppressing caveolin-2 and activating TGF-β pathway

## Data Availability

The data that support the findings of this study are available from the lead corresponding author (Y.L.) on reasonable request.

## References

[1] Wang FS, Fan JG, Zhang Z, Gao B, Wang HY (2014). The global burden of liver disease: the major impact of China. Hepatology.

[2] Pellicoro A, Ramachandran P, Iredale JP, Fallowfield JA (2014). Liver fibrosis and repair: immune regulation of wound healing in a solid organ. Nat. Rev. Immunol..

[3] Lee YA, Wallace MC, Friedman SL (2015). Pathobiology of liver fibrosis: a translational success story. Gut.

[4] Friedman SL (2008). Hepatic stellate cells: protean, multifunctional, and enigmatic cells of the liver. Physiol. Rev..

[5] Gu X, Reagan AM, McClellan ME, Elliott MH (2017). Caveolins and caveolae in ocular physiology and pathophysiology. Prog. Retin. Eye. Res..

[6] Fernandez-Rojo MA, Ramm GA (2016). Caveolin-1 function in liver physiology and disease. Trends Mol. Med..

[7] Bosch M (2011). Caveolin-1 deficiency causes cholesterol-dependent mitochondrial dysfunction and apoptotic susceptibility. Curr. Biol..

[8] Gao L (2014). Caveolin-1 is essential for protecting against binge drinking-induced liver damage through inhibiting reactive nitrogen species. Hepatology.

[9] Fernandez-Rojo MA (2013). Caveolin-1 is necessary for hepatic oxidative lipid metabolism: evidence for crosstalk between caveolin-1 and bile acid signalling. Cell Rep..

[10] Moreno M (2003). Hepatic overexpression of caveolins increases bile salt secretion in mice. Hepatology.

[11] Luo X (2017). Caveolin 1-related autophagy initiated by aldosterone-induced oxidation promotes liver sinusoidal endothelial cells defenestration. Redox Biol..

[12] Mao X (2016). Mechanisms through which hypoxia-induced caveolin-1 drives tumorigenesis and metastasis in hepatocellular carcinoma. Cancer Res..

[13] Peng Y, Croce CM (2016). The role of MicroRNAs in human cancer. Signal Transduct. Target Ther..

[14] Hyun J (2016). MicroRNA-378 limits activation of hepatic stellate cells and liver fibrosis by suppressing Gli3 expression. Nat. Commun..

[15] Ma L (2018). MicroRNA-214 promotes hepatic stellate cell activation and liver fibrosis by suppressing Sufu expression. Cell Death Dis..

[16] Li J (2013). miR-122 regulates collagen production via targeting hepatic stellate cells and suppressing P4HA1 expression. J. Hepatol..

[17] Roderburg C (2013). miR-133a mediates TGF-beta-dependent derepression of collagen synthesis in hepatic stellate cells during liver fibrosis. J. Hepatol..

[18] Tu X (2014). MicroRNA-101 suppresses liver fibrosis by targeting the TGFbeta signalling pathway. J. Pathol..

[19] Ogawa T (2012). MicroRNA-221/222 upregulation indicates the activation of stellate cells and the progression of liver fibrosis. Gut.

[20] Zheng J (2015). Hepatic stellate cell is activated by microRNA-181b via PTEN/Akt pathway. Mol. Cell Biochem..

[21] Lakner AM (2012). Inhibitory effects of microRNA 19b in hepatic stellate cell-mediated fibrogenesis. Hepatology.

[22] Reeves HL, Friedman SL (2002). Activation of hepatic stellate cells—a key issue in liver fibrosis. Front. Biosci..

[23] Shatseva T, Lee DY, Deng Z, Yang BB (2011). MicroRNA miR-199a-3p regulates cell proliferation and survival by targeting caveolin-2. J. Cell Sci..

[24] Liu Y, Jang S, Xie L, Sowa G (2014). Host deficiency in caveolin-2 inhibits lung carcinoma tumour growth by impairing tumour angiogenesis. Cancer Res..

[25] Inagaki Y, Okazaki I (2007). Emerging insights into transforming growth factor beta Smad signal in hepatic fibrogenesis. Gut.

[26] Fabregat I (2016). TGF-beta signalling and liver disease. Febs. J..

[27] Chen YG (2009). Endocytic regulation of TGF-beta signalling. Cell Res..

[28] Lee YB (2009). Twist-1 regulates the miR-199a/214 cluster during development. Nucleic Acids Res..

[29] Lu J, Zhang J, Wang Y, Sun Q (2018). Caveolin-1 scaffolding domain peptides alleviate liver fibrosis by inhibiting TGF-beta1/Smad signalling in mice. Int J. Mol. Sci..

[30] Bray F (2018). Global cancer statistics 2018: GLOBOCAN estimates of incidence and mortality worldwide for 36 cancers in 185 countries. CA Cancer J. Clin..

[31] Mederacke I (2013). Fate tracing reveals hepatic stellate cells as dominant contributors to liver fibrosis independent of its aetiology. Nat. Commun..

[32] Lino Cardenas CL (2013). miR-199a-5p Is upregulated during fibrogenic response to tissue injury and mediates TGFbeta-induced lung fibroblast activation by targeting caveolin-1. PLoS Genet..

[33] Che M (2017). The microRNA-199a/214 cluster targets E-cadherin and claudin-2 and promotes high glucose-induced peritoneal fibrosis. J. Am. Soc. Nephrol..

[34] Chen L, Chen R, Velazquez VM, Brigstock DR (2016). Fibrogenic signaling is suppressed in hepatic stellate cells through targeting of connective tissue growth factor (CCN2) by cellular or exosomal MicroRNA-199a-5p. Am. J. Pathol..

[35] Chen SL, Zheng MH, Yang T, Song M, Chen YP (2013). Disparate profiles of dys-regulated miRNAs in activated hepatic stellate cells. Hepatology.

[36] Lee CG (2012). Farnesoid X receptor protects hepatocytes from injury by repressing miR-199a-3p, which increases levels of LKB1. Gastroenterology.

[37] Roderburg C (2011). Micro-RNA profiling reveals a role for miR-29 in human and murine liver fibrosis. Hepatology.

[38] Racanelli V, Rehermann B (2006). The liver as an immunological organ. Hepatology.

[39] Aranda JF, Canfran-Duque A, Goedeke L, Suarez Y, Fernandez-Hernando C (2015). The miR-199-dynamin regulatory axis controls receptor-mediated endocytosis. J. Cell Sci..

[40] Yin G (2010). TWISTing stemness, inflammation and proliferation of epithelial ovarian cancer cells through MIR199A2/214. Oncogene.

[41] Sakurai K (2011). MicroRNAs miR-199a-5p and -3p target the Brm subunit of SWI/SNF to generate a double-negative feedback loop in a variety of human cancers. Cancer Res..

[42] Uthaya Kumar DB (2016). TLR4 signalling via NANOG cooperates with STAT3 to activate twist1 and promote formation of tumour-initiating stem-like cells in livers of mice. Gastroenterology.

[43] Wang YP (2013). Lipocalin-2 negatively modulates the epithelial-to-mesenchymal transition in hepatocellular carcinoma through the epidermal growth factor (TGF-beta1)/Lcn2/Twist1 pathway. Hepatology.

[44] Helwak A, Kudla G, Dudnakova T, Tollervey D (2013). Mapping the human miRNA interactome by CLASH reveals frequent noncanonical binding. Cell.

[45] Yin S (2016). Differential TGFbeta pathway targeting by miR-122 in humans and mice affects liver cancer metastasis. Nat. Commun..

[46] Yokomori H (2005). High expressions of caveolins on the proliferating bile ductules in primary biliary cirrhosis. World J. Gastroenterol..

[47] Kavsak P (2000). Smad7 binds to Smurf2 to form an E3 ubiquitin ligase that targets the TGFβ receptor for degradation. Mol. Cell.

[48] Razani B (2001). Caveolin-1 regulates transforming growth factor (TGF)-beta/SMAD signalling through an interaction with the TGF-beta type I receptor. J. Biol. Chem..

[49] Weiskirchen R, Gressner AM (2005). Isolation and culture of hepatic stellate cells. Methods Mol. Med..

