# Supplementary material for: Twist1-induced miR-199a-3p promotes liver fibrosis by suppressing caveolin-2 and activating TGF-β pathway
Source: Signal Transduct Target Ther. 2020 Jun 5;5:75. doi: 10.1038/s41392-020-0169-z (PMC7272438; doi:10.1038/s41392-020-0169-z)
Supplement: Supplementary file 1 — Twist1-induced miR-199a-3p promotes liver fibrosis by suppressing caveolin-2 and activating TGF-β pathway [file 41392_2020_169_MOESM1_ESM.docx]

**Supplementary Materials for**

# Twist1-induced miR-199a-3p promotes liver fibrosis by suppressing caveolin-2 and activating TGF-β pathway

Xiaoxue Yang,^1, #^ Liping Ma,^2, 3, #^ Rong Wei,^2^ Tinghong Ye,^1^ JianKang Zhou,^2^ Maoyao Wen,^1^ Ruoting Men,^1^ Rami I. Aqeilan,^4^ Yong Peng,^2, *^ and Li Yang^1, *^

Correspondence to: [yangli_hx@scu.edu.cn](mailto:yangli_hx@scu.edu.cn), [yongpeng@scu.edu.cn](mailto:yongpeng@scu.edu.cn)

**This PDF file includes:**

Materials and Methods

Figures. S1 to S2

Tables S1 to S2

**Materials and Methods**

**Isolation of rat** **hepatocytes**

Normal male Sprague–Dawley rats (Animal Centre of Sichuan university, weighting 300-400g) were used for hepatocytes isolation. All animal studies were approved by the Medical Ethics Committee of the West China Hospital of Sichuan University. Rat hepatocytes were isolated using a two-step collagenase perfusion technique and the cell purity was more than 95%^1^. The rat hepatocytes were grown in High glucose Dulbecco’s modified Eagle medium (DMEM) (Gibco, USA) with 10% fetal bovine serum (FBS) (Biological Industries, Israel). Culture medium was replaced every 48 hours and cells were incubated at 37℃ with 5% CO_2_.

**
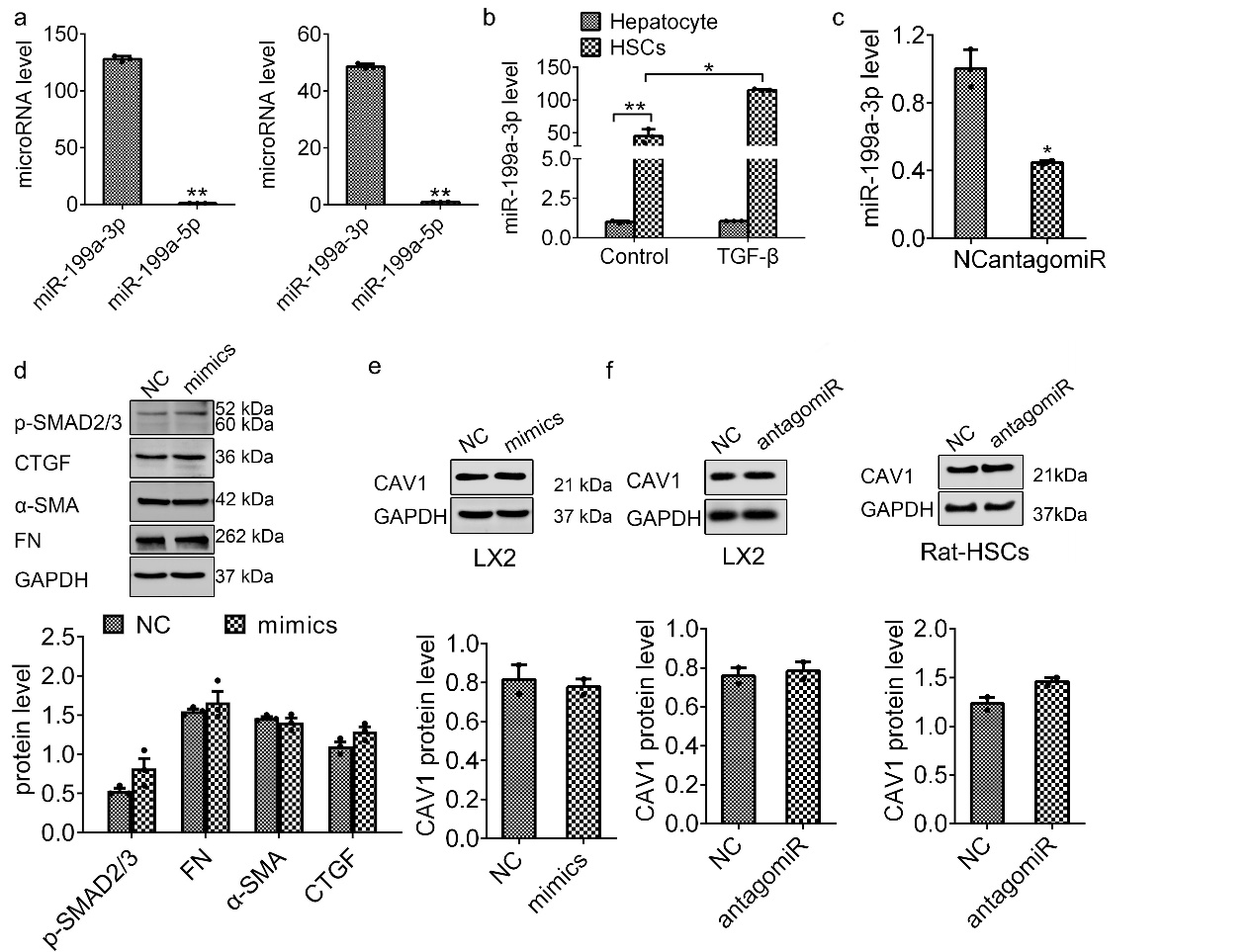
**

**Figure S1. The effect of miR-199a-3p on hepatic fibrosis.** (A) The expression of miR-199a-3p and miR-199a-5p was detected in quiescent rat HSCs (day 1) and activated rat HSCs (day 12). (B) The expression of miR-199a-3p was detected in rat HSCs and hepatocytes with or without TGF-β treatment at 2 ng/ml for 24 h. (C) LX2 cells were transfected with antagomiR-199a-3p for 48 h, and the expression of miR-199a-3p was measured by qRT-PCR. (D) Rat primary HSCs were transfected with miR-199a-3p mimics or NC for 48 h, and the protein levels of FN, p-SMAD2/3, α-SMA and CTGF were examined by Western blotting. (E, F) The protein level of CAV1 after transfection of (E) miR-199a-3p mimics in LX2 cells, and transfection of antagomiR-199a-3p transfection in (F) LX2 cells and rat HSCs, respectively. MiR-199a-3p expression was examined by RT-qPCR analysis and normalized to U6 expression. The relative value of protein band density was measured with Image J software and normalized to GAPDH. Relative expression levels are shown as the means ± SEM obtained from triplicate experiments (unpaired two-sample Student’s *t* test, **P <*0.05 and ***P <* 0.01).

**
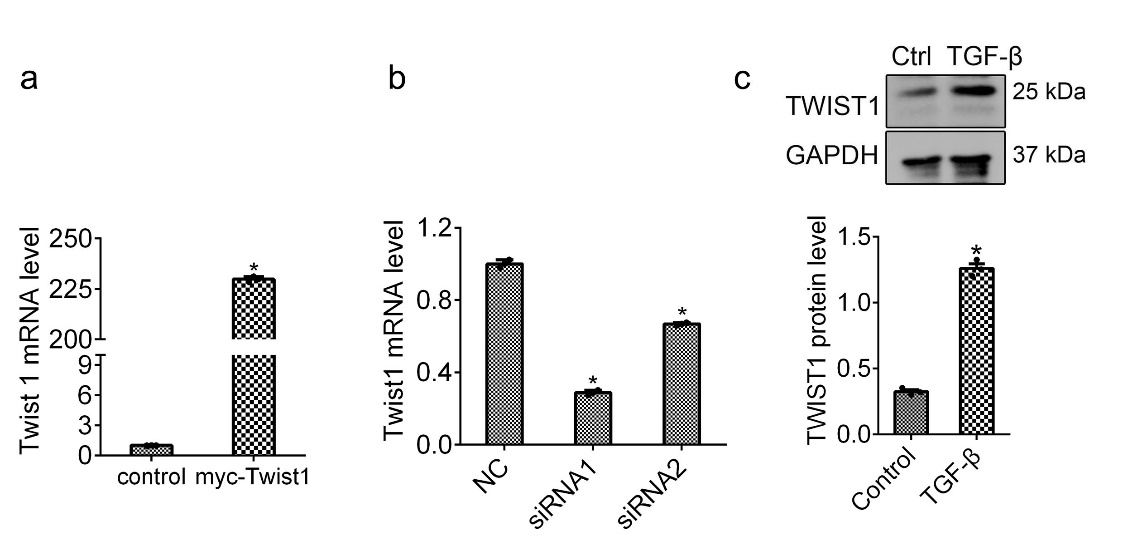
**

**Figure S2. The expression of Twist1 after different treatment.** (A) LX2 cells were infected with Twist1 lentivirus for 72 h, and the *Twist1* mRNA level was examined by qRT-PCR. (B) Rat HSCs were transfected with Twist1 siRNAs for 48 h, and the mRNA of *Twist1* was measured. (C)The protein level of Twist1 after TGF-β treatment in LX2 cells. The mRNA expression was examined by RT-qPCR analysis and normalized to GAPDH expression. The relative value of protein band density was measured with Image J software and normalized to GAPDH. Relative expression levels are shown as the means ± SEM obtained from triplicate experiments (unpaired two-sample Student’s *t* test, **P <*0.05 and ***P <* 0.01).

**Table S1. Primers information for qRT-PCR analysis**

| Primer name | Forward Primer (5'-3') | Reverse Primer (5'-3') |
| --- | --- | --- |
| GAPDH(Rat) | cccatcaccatcttccaggag | gttgtcatggatgaccttggc |
| β-ACTIN(Rat) | actatcggcaatgagcggttc | atgccacaggattccataccc |
| α-SMA(Rat) | ccgagatctcaccgactacc | tccagagcgacatagcacag |
| FN(Rat) | gagagatctggaggtcat | gggtgacacctgagttgaa |
| CTGF(Rat) | cgtgtgcactgccaaagatg | ttgcaactgctttggaagga |
| CAV2(Rat) | tcgactacacagatcctgaga | gaagcctagcttgagatgagag |
| TWIST1(Rat/Human) | actccaagatggcaagctg | tagtgggacgcggacat |
| COL1α1(Rat) | acgtcctggtgaagttggtc | tccagcaataccctgaggtc |
| GAPDH(Human) | agccacatcgctcagacac | gcccaatacgaccaaatcc |
| β-ACTIN(Human) | gtgatctccttctgcatcctga | ccacgaaactaccttcaactcc |
| α-SMA(Human) | ccgagatctcaccgactacc | tccagagcgacatagcacag |
| FN(Human)  CTGF(Human) | gtgttgggaatggtcgtggggaatg  ttagcgtgctcactgacctg | ccaatgccacggccatagcagtagc  ttcacttgccacaagctgtc |
| CAV2(Human) | caccctcagctgtctgcacat | ggcagaaccattaggcaggtctt |
| GAPDH(Mouse) | cgacttcaacagcaactcccactcttcc | tgggtggtccagggtttcttactcctt |
| α-SMA(Mouse) | gtcccagacatcagggagtaa | tcggatacttcagcgtcagga |
| FN(Mouse) | gcaacgtgttatgacgatgg | ctaacggcatgaagcactca |

**Table S2. Primers information for plasmid construction**

| Primer name | Primer (5'-3') |
| --- | --- |
| E-box wt promoter F  E-box wt promoter R  E-box mut promoter F  E-box mut promoter R  myc Twist1 F  myc Twist1 R | tccgctcgaggaagtttgcaggcttgaca  tactaagcttgggctgtgatttccagtctt  gcttaacattacacgaaaacacgtggtaccattttatgcacag  ctgtgcataaaatggtaccacgtgttttcgtgtaatgttaagc  gtcatctagagccaccatggaacaaaaactcatctcagaagaggatctgatgatgcaggacgtgtccatactgaattcctagtgggacgcggacat |

F: forward primer; R: reverse primer; wt: wild type; mut: mutant

**Reference**

1. Yu, F., Chen, B., Dong, P. & Zheng, J. HOTAIR Epigenetically Modulates PTEN

Expression via MicroRNA-29b: A Novel Mechanism in Regulation of Liver Fibrosis.

*Mol Ther.* **25**, 205-217 (2017).
